# Enhancing pathogens detection in suspected geriatric bloodstream infections using Nanopore-targeted sequencing

**DOI:** 10.1128/spectrum.01554-24

**Published:** 2024-11-22

**Authors:** Menghui Zhao, Yan Ouyang, Junchi Mei, Hang Liu

**Affiliations:** 1 Department of Clinical Laboratory,, Institute of Translational Medicine, Renmin Hospital of Wuhan University, Wuhan, China; 2 Medical Research Institute, Frontier Science Center for lmmunology and Metabolism, Wuhan University, Wuhan, China; 3 Department of Nursing, Union Hospital, Tongji Medical College, Huazhong University of Science and Technology, Wuhan Hubei, China; Seton Hall University, South Orange, New Jersey, USA

**Keywords:** bloodstream infections, geriatric patients, Nanopore-targeted sequencing, pathogen detection, blood culture

## Abstract

Bloodstream infections (BSIs) are a critical concern for elderly patients, where prompt and accurate diagnosis is vital for effective treatment. Traditional blood culture methods suffer from delayed results and susceptibility to false-negatives. Nanopore-targeted sequencing (NTS) offers rapid pathogen detection and reporting, presenting a promising alternative. However, the application of NTS for diagnosing suspected BSIs in geriatric patients remains insufficiently studied. We conducted a retrospective cohort study from January 2022 to January 2024, including 198 geriatric patients suspected of BSIs. We utilized NTS to detect pathogen characteristics and compared the effectiveness and consistency of NTS with simultaneous blood culture testing for pathogen detection. NTS demonstrated a pathogen detection rate of 61.1%. The most common bacterial pathogens were *Escherichia coli* and *Staphylococcus aureus* (each 7.7%), while *Candida albicans* was the most prevalent fungal pathogen (30%). Mixed-bacterial infections were detected in 21.7% of NTS-positive cases, and concurrent bacterial–fungal infections were observed in 9.92% of these cases. NTS-positive patients had higher rates of comorbidities, elevated inflammatory markers, and worse prognoses compared to NTS-negative patients. NTS exhibited a significantly higher pathogen detection rate and faster turnaround time than blood culture (78.1% vs 42.2%, *P* < 0.001), with an agreement rate of 65.6%. The elderly BSI patients frequently involve multiple or mixed infections and correlate with poorer prognoses. NTS provides a faster and more sensitive diagnostic alternative to traditional blood culture, potentially improving clinical outcomes and guiding more effective treatment strategies. This study highlights the need for further research to validate the routine clinical integration of NTS for managing BSIs in geriatric populations.

**IMPORTANCE:**

Bloodstream infections (BSIs) in elderly patients pose substantial diagnostic and therapeutic challenges due to the limitations of traditional blood culture methods, which are hampered by slow turnaround times and false-negatives. Nanopore-targeted sequencing (NTS) emerges as a significant advancement, offering rapid and accurate pathogen detection directly from blood samples. This study demonstrates that NTS provides a higher detection rate and faster results than conventional blood cultures, crucial for the timely management of BSIs in geriatric patients, who often present with multiple or mixed infections and have poorer clinical outcomes. The findings underscore the potential of NTS to enhance diagnostic accuracy and speed, informing more effective treatment strategies and improving overall patient outcomes. Further research is essential to establish NTS as a routine diagnostic tool in the clinical management of BSIs in the elderly.

## INTRODUCTION

The aging population is growing at an unprecedented rate, presenting unique and pressing challenges to healthcare systems worldwide (1). Among these challenges, infectious diseases, particularly bloodstream infections (BSIs), pose significant health threats to the elderly population (2–4). Over 50% of BSIs occur in individuals aged 65 years and older, leading to severe complications such as sepsis, multi-organ failure, and increased mortality (5–7). This heightened vulnerability among the elderly can be attributed to age-related immune senescence, which impairs their ability to combat infections, as well as the high prevalence of chronic comorbidities such as diabetes, hypertension, and heart disease (8, 9). Additionally, age-related physiological changes, such as diminished renal function and altered drug metabolism, would further influence the progression of infections and the efficacy of therapeutic interventions (10). Identifying the etiological pathogen is crucial for diagnosing BSIs. However, there remains a lack of rapid, sensitive, and accurate diagnostic approaches to detect these pathogens.

Traditional blood cultures, the gold standard for diagnosing BSI pathogens, often take several days to yield results and may show reduced sensitivity, particularly in detecting fastidious or slow-growing organisms (11). This delay, coupled with immunosenescence-induced attenuation and atypical immune responses in the elderly, compromises the effectiveness of conventional diagnostic methods, potentially leading to delayed or inappropriate treatments, extended hospital stays, and higher mortality. Recent advances in sequencing technologies, especially Nanopore-targeted sequencing (NTS), offer promising solutions by enabling rapid, comprehensive, and highly sensitive detection of pathogens directly from bronchoalveolar-lavage fluid, blood, and urine samples, circumventing the need for culture (12–15). NTS can simultaneously identify a broad spectrum of bacterial and fungal pathogens, even in cases of low pathogen loads or mixed infections, thus proving particularly advantageous for the elderly where timely therapeutic intervention is critical. However, despite the progress of NTS in diagnosing other infections, there remains a notable research gap in its application for detecting BSI pathogens, specifically in the elderly population.

This study aims to evaluate the applicability and effectiveness of NTS in identifying BSI pathogens in older adults. We will examine infection characteristics and pathogen distribution using NTS and compare NTS with traditional blood culture methods to assess its consistency within this demographic. Our findings indicate that NTS effectively detects BSIs in the elderly, particularly in cases with high rates of multiple or mixed infections. NTS surpasses traditional blood cultures in pathogen detection, improves detection rates, and reduces diagnostic turnaround time, facilitating timely and personalized therapeutic interventions. The study’s findings are expected to advance diagnostic practices for BSIs in the elderly, improve clinical decision-making, and ultimately enhance patient outcomes through the application of NTS approaches.

## MATERIALS AND METHODS

### Patients and specimens

This retrospective cohort study included 198 geriatric patients hospitalized with suspected BSIs at the Department of Geriatrics, Renmin Hospital of Wuhan University, from January 2022 to January 2024. While the data collection and analysis were retrospective, the samples were processed in real-time upon collection, with both NTS and traditional blood culture performed immediately to preserve nucleic acid integrity and pathogen viability. The inclusion criteria were as follows: patients aged over 60 years presenting with clinical signs indicative of BSIs, such as fever (>38°C) or hypothermia (< 36°C), chills, hypotension, altered mental status, or systemic inflammatory response syndrome; and laboratory indicators of infection, including altered white blood cell counts, elevated inflammatory markers such as C-reactive protein (CRP) or procalcitonin (PCT), or positive blood cultures. Exclusion criteria included pre-existing BSIs at the time of hospitalization, advanced cancer, or any terminal-stage disease. Patient data were extracted from the hospital’s medical record database including NTS detection reports and medical records.

This study adhered to the guidelines of the Declaration of Helsinki and received approval from the Institutional Review Board of Renmin Hospital of Wuhan University. Given the retrospective design of this study and the absence of patient privacy concerns, the Ethics Committee of Renmin Hospital of Wuhan University waived the requirement for informed consent (No. WDRY2022-K157).

### NTS methodology and bioinformatics methodology

NTS was conducted using the Oxford Nanopore MinION device within 24 hours post-sample collection, adhering to established protocols for library preparation and sequencing as previously described (14, 16). The standardized procedures for primer design, NTS amplification, sequence alignment, and bioinformatics analysis were supplied by DGENSEE (Wuhan, China) and are thoroughly described in the supplementary methods section. Briefly, DNA was extracted from whole blood samples using a commercial DNA extraction kit (product number S1006; Sansure, Changsha, China), followed by quantification and quality assessment. Sequencing libraries were generated through a transposase-based method, and sequencing proceeded until sufficient coverage was achieved. Universal NTS primers designed in-house targeted the entire 16S rRNA gene of bacteria, the whole internal transcribed spacer (ITS) gene of fungi, and a segment of the rpoB gene of *Mycobacterium* spp.

Bioinformatics analysis involved base-calling via Guppy software (version 3.1.5), followed by mapping clean reads to 16S rDNA, ITS, and rpoB reference genes from the National Center for Biotechnology Information database for taxonomic classification. Medaka software (version 0.10.1) was used to generate a consensus sequence of pathogen reads assigned to the same species, which was then remapped to the reference database to detect species-level taxonomy as the final result.

### Blood Cultures and Detection of Clinical Infection Indexes

Blood cultures were performed using automated systems (BacT/ALERT, bioMérieux), with positive cultures further analyzed by matrix-assisted laser desorption ionization–time-of-flight mass spectrometry (MALDI-TOF MS; Vitek MS; bioMérieux, France). Antimicrobial susceptibility testing was carried out using the BD Phoenix 100 system (Becton, Dickinson and Company, Sparks, MD, USA).

Blood cell counts were conducted with the XN-9000 Sysmex hematology analyzer (Sysmex, Kobe, Japan), following the manufacturer’s protocols. CRP and serum amyloid A (SAA) were assessed using an automatic protein analyzer H780-3 (Xilaiheng, Shenzhen, China). Serum PCT levels were determined using the Cobas e801, an automated chemiluminescence immunoassay analyzer (Roche, Mannheim, Germany). All laboratory tests were conducted in accordance with the routine clinical laboratory procedures of Renmin Hospital of Wuhan University.

### Statistical analysis

In our study, comparative analyses of pathogen detection rates, agreement rates, and laboratory turnaround times between NTS and blood cultures were conducted. Continuous variables, such as laboratory variables and turnaround times, were expressed as medians with interquartile ranges (IQR) and compared using the Mann–Whitney U test due to the non-normal distribution of the data. Categorical variables, including pathogen detection rates and agreement rates, were expressed as percentages and compared using the χ test or Fisher’s exact test, depending on the sample size greater or less than 5. A *p* value of < 0.05 was considered statistically significant.

## RESULTS

### Characteristics of Geriatric Patients with Suspected BSIs

**Fig 1:**
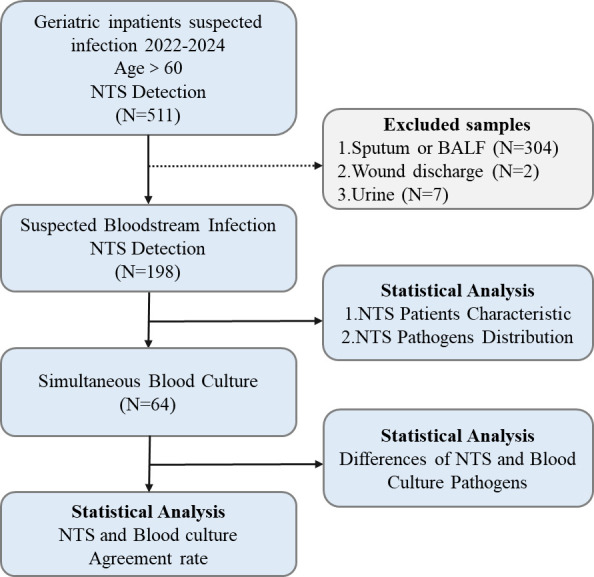
Flowchart of suspected geriatric bloodstream infection selection. Note: BALF, bronchoalveolar lavage.

The study cohort comprised 198 geriatric patients with suspected BSIs (Fig. 1). Among these patients, 73.7% were male, with a median age of 90 years (IQR: 84–93). The most prevalent comorbidities included hypertension in 47.8% of the patients, heart disease in 34.7%, and diabetes in 24.7%. Laboratory analyses revealed a median white blood cell (WBC) count of 8.1 × 10^9^ /L (IQR: 5.9–10.7 × 10^9^ /L), a median neutrophil count (Neu) of 6.0 × 10^9^ /L (IQR: 3.8–8.8 × 10^9^ /L), and a median neutrophil percentage of 75.1% (IQR: 63.6%-83.8%). The CRP levels had a median of 31.9 mg/L (IQR: 12.0–67.0 mg/L), while the PCT levels showed a median of 0.17 ng/mL (IQR: 0.08–0.84 ng/mL), and the serum SAA levels had a median of 176.6 mg/L (IQR: 45.2–300 mg/L). These findings underscore the elevated inflammatory markers typically observed in patients with suspected BSIs, highlighting the systemic inflammatory response within the elderly population (Table 1).

**TABLE 1:** Baseline characteristics and laboratory variables of suspected bloodstream infection patients^*a*^^, *b*^

Characteristics	Total group (*n* = 198)	Subgroup with concurrent NTS and culture testing (*n* = 64)
Male (n, %)	146 (73.7)	50 (78.1)
Female (n, %)	52 (26.3)	14 (21.9)
Age (years)	90 (84–93)	90 (82–93)
Hypertension (n, %)	94 (47.8)	34 (53.1)
Diabetes (n, %)	49 (24.7)	17 (26.6)
Heart disease (n, %)	74 (37.4)	30 (46.9)
Laboratory variables		
WBC (x10^9^/L)	8.1 (5.9–10.7)	8.07 (6.0–11.4)
Neu (x10^9^/L)	6.0 (3.8–8.8)	5.68 (3.6–9.5)
Neu percent (%)	75.1 (63.6–83.8)	76.27 (61.2–85.0)
CRP (mg/L)	31.9 (12.0–67.0)	31.9 (14.5–72.0)
PCT (ng/mL)	0.17 (0.08–0.84)	0.19 (0.09–1.14)
SAA (mg/L)	176.6 (45.2–300)	172.0 (53.0–300)

^*a*^ Categorical variables were presented with numbers (percentages). Continuous variables were presented with mean ± standard deviation or median (25th percentile–75th percentile), depending on whether the data conform to a normal distribution.

^*b*^ WBC, white blood cell; Neu, neutrophil; CRP, C-reactive protein; PCT, procalcitonin; SAA, serum amyloid A; heart disease including atrial fibrillation, valvular disease, or coronary heart disease.

### Pathogen Detection by NTS

**Fig 2:**
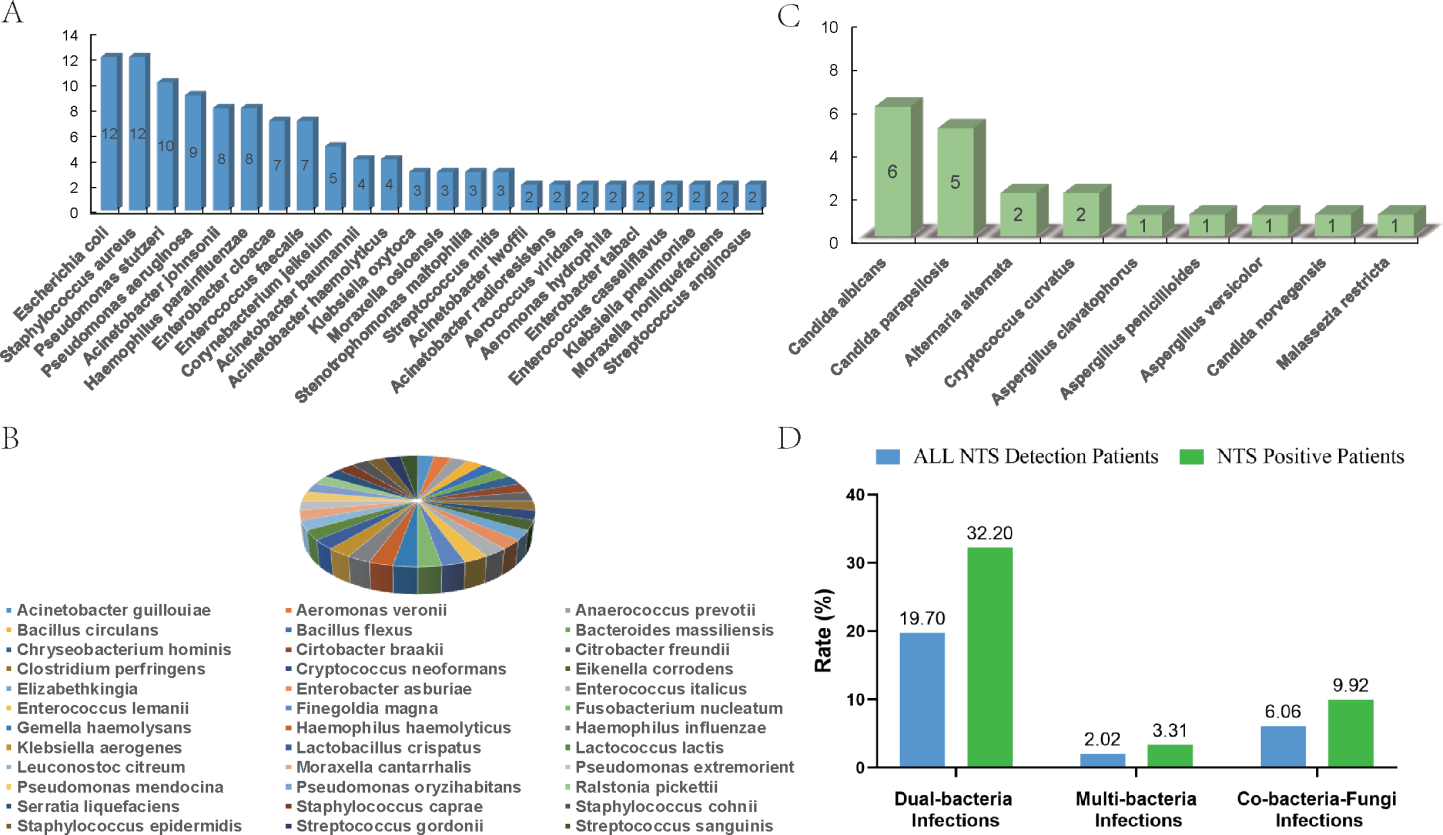
Pathogen distribution characteristic of NTS. (**A).** Distribution of bacterial pathogens detected by NTS; (B) frequency of specific bacterial species; distribution of fungal pathogens detected by NTS. D. Prevalence of mixed bacterial and fungal infections.

NTS demonstrated a pathogen detection rate of 61.1% (121/198) in whole suspected BSI geriatric patients. The most prevalent bacterial pathogens identified were *Escherichia coli* and *Staphylococcus aureus* (*n* = 12, 7.7% each), followed by *Pseudomonas stutzeri* (*n* = 10, 6.4%), *Pseudomonas aeruginosa* (*n* = 9, 5.8%), *Acinetobacter johnsonii* and *Haemophilus parainfluenzae* (*n* = 8, 5.1% each), *Enterobacter cloacae* and *Enterococcus faecalis* (*n* = 7, 4.49% each), *Corynebacterium jeikeium* (*n* = 5, 3.2%), and both *Acinetobacter baumannii* and *Acinetobacter haemolyticus* (*n* = 4, 2.6% each). Additionally, several rare or difficult-to-culture bacteria were also detected, such as *Moraxella nonliquefaciens* (*n* = 2, 1.3%), *Fusobacterium nucleatum*, *Eikenella corrodens*, *Aerococcus viridans*, *Enterobacter tabaci*, and *Elizabethkingia* (*n* = 1, 0.7% each) (Fig. 2A and B). Among fungal pathogens, *Candida albicans* (*n* = 6, 30%) and *Candida parapsilosis* (*n* = 5, 25%) were most common, followed by *Alternaria alternata* and *Cryptococcus curvatus* (*n* = 2, 10% each) and *Aspergillus clavatophorus*, *Aspergillus penicillioides*, *Aspergillus versicolor*, and *Candida norvegensis* (*n* = 1, 5% each) (Fig. 2C). Notably, dual and multibacterial infections were identified in 21.72% of all NTS-detected patients and 35.54% of all NTS-positive patients. Additionally, concurrent bacterial–fungal infections accounted for 9.92% of all NTS-positive cases (Fig. 2D). These results highlight a significant prevalence of multiple and mixed infections among elderly patients presenting with suspected BSIs.

### Comparison of Pathogen Detection Rates and Laboratory Turnaround Times in the Concurrent NTS and Culture Testing Subgroup

In a comparative analysis of 64 samples subjected simultaneously to NTS and traditional blood culture (Table 2), NTS exhibited a significantly higher overall pathogen detection rate compared to blood culture (78.1% vs 42.2%, *P* < 0.001). The agreement rate of 65.6% indicates substantial concordance between NTS and blood culture results. Specifically, NTS outperformed blood culture in detecting bacterial pathogens (76.6% vs 39.1%, *P* < 0.001). Compared to blood cultures, NTS demonstrated a higher positive rate for detecting fungal pathogens (15.6% vs 9.4%), although the difference in detection rates was not statistically significant (*P* = 0.285).

**TABLE 2:** Comparison of pathogen detection rates and laboratory turnaround times Using NTS and culture testing (*n* = 64)

Characteristic	NTS	Culture	Agreement rate (%)	P
Pathogen-positive (n, %)	50 (78.1)	27 (42.2)	65.6	<0.001
Bacteria-positive (n, %)	49 (76.6)	25 (39.1)	68.8	<0.001
Fungi-positive (n, %)	10 (15.6)	6 (9.4)	87.5	0.285
Mixed bacterial infection (n, %)	20 (31.3)	2 (3.1)	34.4	<0.001
Co-bacteria and fungi infections (n, %)	9 (14.1)	2 (3.1)	84.4	0.027
Laboratory turnaround time (day)	2 (1, 2)	5 (4, 6)	-	<0.001

^*a*^ Categorical variables were presented with numbers (percentages). Continuous variables were presented with mean ± standard deviation or median (25th percentile–75th percentile), depending on whether the data conform to a normal distribution.

^*b*^ "-", not available.

NTS also significantly surpassed blood culture in detecting mixed bacterial infections (31.3% vs 3.1%, *P* < 0.001), indicating NTS’s superior capability in identifying complex bacterial infections. Furthermore, NTS was more effective in concurrent bacterial and fungal infections compared to blood culture (14.1% vs 3.1%, *P* = 0.027). NTS provided significantly faster laboratory turnaround times than blood culture, with a median of 2 days versus 5 days, respectively (*P* < 0.001), reflecting NTS’s efficiency in delivering timely diagnostic results.

### Differences Between NTS-Negative and NTS-Positive Groups in Whole Suspected BSI Patients

There were no significant differences in gender distribution or median age between the whole NTS-negative and NTS-positive groups (*P* = 0.797 and *P* = 0.968, respectively). Hypertension was more prevalent in the NTS-positive group compared to the NTS-negative group (57.9% vs 31.2%, *P* < 0.001). Diabetes was also more common in the NTS-positive group (29.8% vs 16.9%, *P* = 0.041), as well as heart disease (44.6% vs 26.0%, *P* = 0.008).(Table 3)

**TABLE 3:** Characteristic difference between the NTS-negative and positive groups in suspected bloodstream infection patients^*a*^^, *b*^

Characteristics	NTS-negative (*n* = 77)	NTS-positive (*n* = 121)	P
Male (n, %)	56 (72.7)	90 (74.4)	0.797
Female (n, %)	21 (27.3)	31 (25.6)	
Age (years)	89 (82–93)	90 (85–92)	0.968
Hypertension (n, %)	24 (31.2)	70 (57.9)	<0.001
Diabetes (n, %)	13 (16.9)	36 (29.8)	0.041
Heart disease (n, %)	20 (26.0)	54 (44.6)	0.008
Laboratory variables			
WBC (x10^9^/L)	6.6 (5.2–9.5)	9.1 (6.5–11.6)	<0.001
Neu (x10^9^/L)	4.8 (3.2–7.0)	6.9 (4.4–9.7)	<0.001
Neu percent (%)	70.5 (60.0–77.1)	78.4 (67.0–86.8)	<0.001
CRP (mg/L)	26.8 (7.4–53.6)	33.8 (14.9–80.1)	0.010
PCT (ng/mL)	0.13 (0.06–0.62)	0.25 (0.10–1.08)	0.015
SAA (mg/L)	148 (29.1–300)	207 (47.6–300)	0.184
Clinical Outcomes			
In-hospital mortality (n, %)	5 (6.5)	19 (15.7)	0.053
Functional status deterioration (n, %)	7 (9.1)	24 (19.8)	0.043

^*a*^ Categorical variables were presented with numbers (percentages). Continuous variables were presented with mean ± standard deviation or median (25th percentile–75th percentile), depending on whether the data conform to a normal distribution.

^*b*^ WBC, white blood cell; Neu, neutrophil; CRP, C-reactive protein; PCT, procalcitonin; SAA, serum amyloid A; heart disease including atrial fibrillation, valvular disease, or coronary heart disease.

Laboratory findings indicated higher median WBC counts in the NTS-positive group (9.1 × 10^9^ /L, IQR: 6.5–11.6) compared to the NTS-negative group (6.6 × 10^9^ /L, IQR: 5.2–9.5) (*P* < 0.001). Neutrophil counts were also elevated in the NTS-positive group (median: 6.9 × 10^9^ /L, IQR: 4.4–9.7) versus the NTS-negative group (median: 4.8 × 10^9^ /L, IQR: 3.2–7.0) (*P* < 0.001), along with neutrophil percentages (median: 78.4%, IQR: 67.0%–86.8% vs 70.5%, IQR: 60.0%–77.1%) (*P* < 0.001). CRP levels were higher in the NTS-positive group (median: 33.8 mg/L, IQR: 14.9–80.1) compared to the NTS-negative group (median: 26.8 mg/L, IQR: 7.4–53.6) (*P* = 0.01). Similarly, PCT levels were elevated in the NTS-positive group (median: 0.25 ng/mL, IQR: 0.10–1.08) compared to the NTS-negative group (median: 0.13 ng/mL, IQR: 0.06–0.62) (*P* = 0.015). No significant difference was observed in SAA levels between the groups (*P* = 0.184).(Table 3)

In-hospital mortality was also higher in the NTS-positive group compared to the NTS-negative group (15.7% vs 6.5%), approaching statistical significance (*P* = 0.053). Additionally, functional status deterioration was significantly greater in the NTS-positive group versus the NTS-negative group (19.8% vs 9.1%, *P* = 0.043), which indicated patients with positive NTS results exhibited worse clinical outcomes.(Table 3)

### Comparison of Characteristics and Pathogen Detection Rates between Male and Female Groups in Suspected BSI Patients

To investigate potential sex differences in pathogen detection rates, we divided the patient cohort into male and female groups (Table 4). Males exhibited a significantly higher prevalence of hypertension (54.1% vs 30.1%, *P* = 0.002) and diabetes (28.8% vs 9.6%, *P* = 0.003) compared to females. Additionally, CRP levels were slightly higher in males than in females (33.8 mg/L vs 25.4 mg/L, *P* = 0.044), suggesting a possible elevated inflammatory response among males. Regarding pathogen detection rates, males demonstrated a significantly higher overall detection rate than females (65.8% vs 48.1%, *P* = 0.025). Bacterial infections were more commonly detected in males than in females (63.0% vs 44.2%, *P* = 0.018), whereas fungal infection rates showed no statistically significant difference (11.6% in males vs 7.7% in females, *P* = 0.427). Additionally, mixed bacterial infections and co-infections involving both bacteria and fungi were observed more frequently in males; however, these differences did not reach statistical significance. In terms of clinical outcomes, males had a higher in-hospital mortality rate (14.4% vs 5.8%, *P* = 0.102) and more common functional status deterioration (17.8% vs 9.6%, *P* = 0.163) compared to females, though these differences did not reach statistical significance.

**TABLE 4:** Comparison of characteristics between male and female groups in suspected bloodstream infection patients

Characteristics	Male (*n* = 146)	Female (*n* = 52)	P
Age (years)	90 (85–93)	88 (81–93)	0.351
Hypertension (n, %)	79 (54.1)	15 (28.8)	0.002
Diabetes (n, %)	44 (30.1)	5 (9.6)	0.003
Heart disease (n, %)	59 (40.4)	15 (28.8)	0.139
Laboratory variables			
WBC (x10^9^/L)	8.34 (6.0–11.3)	7.48 (5.7–10.0)	0.222
Neu (x10^9^/L)	6.1 (3.8–8.9)	5.56 (3.8–7.8)	0.374
Neu percent (%)	75.0 (64.0–83.2)	75.7 (63.7–84.3)	0.774
CRP (mg/L)	33.8 (14.7–71.9)	25.4 (4.47–57.3)	0.044
PCT (ng/mL)	0.15 (0.07–1.05)	0.13 (0.06–1.03)	0.679
SAA (mg/L)	209 (55.3–300)	141 (28.4–300)	0.099
NTS pathogen detection			
Pathogen-positive (n, %)	96 (65.8)	25 (48.1)	0.025
Bacteria-positive (n, %)	92 (63.0)	23 (44.2)	0.018
Fungi-positive (n, %)	17 (11.6)	4 (7.7)	0.427
Mixed-bacterial infection (n, %)	31 (21.2)	7 (13.5)	0.222
Co-bacteria and fungi infections (n, %)	11 (7.5)	4 (7.7)	1.000^a^
Clinical outcomes			
In-hospital mortality (n, %)	21 (14.4)	3 (5.8)	0.102
Functional status deterioration (n, %)	26 (17.8)	5 (9.6)	0.163

^*a*^ a, Fisher’s exact test used due to smaller expected frequencies. WBC, white blood cell; Neu, neutrophil; CRP, C-reactive protein; PCT, procalcitonin; SAA, serum amyloid A; heart disease including atrial fibrillation, valvular disease, or coronary heart disease.

### Clinical Characteristics, Pathogen Profiles, Treatments, and Outcomes in Patients with Co-bacterial and Fungal Infections

To explore the comprehensive detection, treatment, and prognostic outcomes associated with mixed infections using NTS, Table 5 presents a detailed account of clinical characteristics, pathogen profiles, therapeutic interventions, and clinical outcomes observed in a cohort of 12 elderly patients (aged 68–98 years). These patients were diagnosed with concurrent bacterial and fungal infections following suspicion of BSIs, highlighting the utility of NTS in identifying complex infectious etiologies in geriatric populations.

**TABLE 5:** Clinical characteristics, pathogen profiles, treatments, and outcomes in patients with co-bacterial and fungal infections

Patient ID	Age/sex	Pathogen(s) detected by NTS (read number)	Blood culture	Treatment	Outcomes
7	89/male	*Enterococcus casseliflavus* (98) *Candida metapsilosis* (496)	Not available	Imipenem; vancomycin	Death
49	90/male	*Aeromonas veronii* (1,268) *Candida parapsilosis* (1,562)	Negative	Colistin; micafungin	Recover
75	86/male	*Enterococcus faecium* (23,113) *Candida parapsilosis* (428)	*Enterococcus faecium*	Linezolid, imipenem, and voriconazole	Recover
97	87/female	*Pseudomonas extremorientalis* (612) *Staphylococcus epidermidis* (210) *Candida albicans* (12,344)	*Staphylococcus epidermidis;* *Candida albicans*	Linezolid, imipenem, and micafungin	Death
109	92/male	*Enterobacter cloacae* (234) *Candida norvegensis* (16390)	*Enterobacter cloacae* *Candida norvegensis*	Meropenem; micafungin	Death
132	87/male	*Acinetobacter baumannii* (116) *Candida albicans* (474)	*Candida albicans*	Imipenem; micafungin	Deterioration
134	98/female	*Enterococcus faecalis* (13,886) *Alternaria alternat*a (922)	*Enterococcus faecalis*	Levofloxacin; micafungin	Death
146	*94/female*	*Enterococcus faecium* (4789) *Malassezia restricta* (575)	*Enterococcus faecium*	Teicoplanin, imipenem, and voriconazole	Death
150	90/male	*Escherichia coli* (165) *Enterobacter tabaci* (26) *Aspergillus versicolor* (14,106)	Negative	Linezolid, imipenem, and micafungin	Death
165	92/male	*Staphylococcus aureus* (169) *Candida parapsilosis* (2,830)	*Candida parapsilosis*	Linezolid; micafungin	Deterioration
183	90/male	*Staphylococcus aureus* (36) *Enterobacter asburiae* (23) *Alternaria alternat*a (541)	Negative	Teicoplanin, moxifloxacin, and micafungin	Death
189	68/male	*Staphylococcus aureus* (25,991) *Candida albicans* (13,012)	*Staphylococcus aureus* *Candida albicans*	Linezolid; voriconazole	Deterioration

The cohort under study, predominantly male, exhibited a spectrum of mixed-pathogen infections, encompassing prevalent bacteria like *Enterococcus faecium* and *Staphylococcus aureus*, as well as fungi such as *Candida albicans* and *Candida parapsilosis*. Comparison with blood cultures confirmed the presence of some pathogens identified by NTS, emphasizing its broader detection capabilities for mixed-pathogen infections.

Despite aggressive treatment regimens involving combinations of antibiotics and antifungals, such as linezolid, carbapenems, micafungin, and voriconazole, clinical outcomes were predominantly unfavorable in this cohort of patients with co-bacterial and fungal infections. Specifically, seven patients succumbed to their infections, three experienced clinical deterioration, and only two patients showed signs of recovery. These high mortality and deterioration rates underscore the critical nature of BSIs in the geriatric population, highlighting the urgent need for early and precise pathogen detection to effectively manage co-infections in BSIs.

## DISCUSSION

Identifying the etiological pathogen is crucial for diagnosing BSIs; however, conventional blood culture methods often lack sensitivity and have prolonged turnaround times, thereby delaying critical therapeutic decisions (7). NTS offers a novel approach for rapid and thorough pathogen detection directly from clinical samples. We focus on a cohort of 198 geriatric patients suspected of BSIs, utilizing NTS as the primary pathogen detection method. The predominance of male patients and a median age of 90 years underscore the vulnerability of elderly males to BSIs, consistent with previous findings identifying advanced age and male gender as risk factors for severe infections (5, 7, 17). The significantly higher pathogen and bacterial infection detection rates in males support prior research, indicating that males may be more prone to bacterial infections, possibly due to immune system variations and hormonal influences (18, 19). The high prevalence of comorbidities in this cohort reflects the multi-morbidity burden typical among the elderly, complicating infection management. Previous research highlights the susceptibility of elderly individuals to severe infections due to compromised immune function and systemic inflammatory responses (20). Laboratory findings, including elevated inflammatory markers such as WBC counts, neutrophil percentages, CRP, and PCT levels, indicate robust and typical systemic inflammatory profiles in these elderly patients with BSIs.

The spectrum of bacterial pathogens identified by NTS, including *Escherichia coli*, *Staphylococcus aureus*, and *Pseudomonas* spp., aligns with pathogen profiles documented in previous studies (5, 21). Furthermore, NTS proved capable of detecting rare and fastidious pathogens such as the anaerobic bacterium *Fusobacterium nucleatum* and the nonfermenting bacterium *Elizabethkingia* spp. , both of which were significantly associated with increased mortality, thereby underlining the potential of NTS to enhance diagnostic accuracy and improve patient outcomes in the elderly (22, 23). In line with previous research, our findings showed that NTS effectively detected common fungal pathogens in BSIs, particularly *Candida* spp. and *Aspergillus* spp (24). Interestingly, our study is the first to detect *Alternaria alternata* in BSI patients using NTS, a pathogen typically related to a poor prognosis of asthma, although further confirmation is required (25).

Our results indicated that NTS-positive patients exhibited higher rates of comorbidities, elevated inflammatory markers, and worse prognoses than NTS-negative patients, suggesting that NTS-positive individuals are more likely to experience severe infections and systemic inflammation and that the presence of comorbidities alongside BSIs would complicate treatment options. Notably, NTS revealed a 9.92% incidence of mixed bacterial and fungal infections and a 32.2% occurrence of polymicrobial infections among elderly BSI patients. Li *et al*. found that mixed *Candida albicans* and bacterial BSIs were associated with prolonged mechanical ventilation and extended ICU stays compared to monomicrobial *Candida albicans* BSIs in adult patients (26). Furthermore, patients identified by NTS with co-bacterial and fungal infections demonstrated particularly poor outcomes, with over 80% in-hospital mortality and clinical deterioration despite broad-spectrum antimicrobial and antifungal treatments. One explanation is that advancing age is associated with a higher incidence of gastrointestinal disorders and reduced renal function, which can significantly alter drug metabolism and consequently affect the efficacy of therapeutic interventions (27).These findings emphasize the urgent need for the clinical implementation of NTS to refine pathogen early and accurate diagnostic strategies and guide appropriate treatment for enhancing survival outcomes for elderly patients, particularly in those with bacterial–fungal co-infections who require additional focused care.

The total pathogen detection rate achieved by NTS in this study was higher than previously reported rates based on conventional blood culture methods for BSIs (6). Furthermore, NTS showed a markedly higher positive detection rate and faster turnaround time in our comparative analysis with simultaneous blood culture testing. Currently, blood culture remains the established gold standard for identifying pathogens in BSIs; however, it has inherent limitations, such as relatively lengthy culture cycles and challenges in isolating fastidious or slow-growing pathogens. Following the COVID-19 pandemic, pathogen detection methods have proliferated, providing expanded options for clinical diagnostics, especially for PCR-based technologies.

mNGS excels at comprehensively detecting a broad spectrum of pathogens, including uncommon, novel, and polymicrobial infections involving bacteria, fungi, and viruses, without needing prior knowledge of the infection characteristics (28). However, its operational complexity, extended sequencing time, high costs, and the need for batch testing due to sample accumulation requirements present significant challenges (29). NTS achieves diagnostic performance comparable to that of mNGS, while offering advantages such as automated nucleic acid isolation, fast sequencing library setup, and synchronous analytical processing. These enabled the testing and analysis of 95% of samples within approximately 6–7 hours, facilitating early diagnosis, particularly for elderly patients with suspected BSIs, and providing a timely treatment in urgent clinical scenarios (14, 30). Rapid pathogen identification can lead to earlier initiation of targeted therapy, potentially improving outcomes in geriatric patients, who often have less physiological reserve and are at higher risk for complications from BSIs.

Our study has several limitations. First, its relatively small sample size and single-center design may restrict the broader applicability of our findings. Recent research suggests that NTS is comparable to mNGS in pathogen detection and offers advantages over traditional blood culture (30). Second, NTS relies on primer amplification designed for specific pathogens, and its effectiveness across diverse pathogens remains uncertain, potentially resulting in the oversight of novel or uncommonly characterized pathogens. Additionally, certain rare bacteria detected by NTS may not be confirmed by blood culture, and effectively excluding contaminating bacteria can be challenging. Positive blood cultures are essential for diagnosing pathogen infections by confirming the presence of clinically relevant microorganisms and adhering to Koch’s postulates. Our emphasis on using sterile blood samples helps mitigate potential contaminations to some extent. Lastly, our current NTS panel cannot detect viral infections or identify drug-resistant genes, but recent advancements suggest the potential for detecting RNA viruses and the *Klebsiella pneumoniae* carbapenemase gene (31–33). Addressing these limitations would enhance the practical application of NTS in elderly patients with BSIs, improving diagnostic accuracy, reducing detection times, and ultimately enhancing patient outcomes.

### Conclusion

In conclusion, NTS demonstrated superior pathogen detection capabilities compared to traditional blood culture in a geriatric population with suspected BSIs, particularly for bacterial and mixed infections. The rapid turnaround time and broad-spectrum detection of NTS provide significant clinical advantages, especially in managing complex infections in the elderly. These findings highlight the potential of NTS to enhance the diagnostic accuracy and improve clinical outcomes in geriatric patients with suspected BSIs. Further research is needed to integrate NTS into routine clinical workflows and to explore its impact on patient management and healthcare outcomes.

## Data Availability

The data used and/or analyzed during the current study are available from the corresponding author on reasonable request.

## SUPPLEMENTAL MATERIAL

Supplemental material spectrum.01554-24-s0001.docx Supplement methods.

Table S1 spectrum.01554-24-s0002.docx Primers for NTS used in this study.

ASM does not own the copyrights to Supplemental Material that may be linked to, or accessed through, an article. The authors have granted ASM a non-exclusive, world-wide license to publish the Supplemental Material files. Please contact the corresponding author directly for reuse.
