# Supplementary material for: Enhancing pathogens detection in suspected geriatric bloodstream infections using Nanopore-targeted sequencing
Source: Microbiol Spectr. 2024 Nov 22;13(1):e01554-24. doi: 10.1128/spectrum.01554-24 (PMC11705817; doi:10.1128/spectrum.01554-24)
Supplement: Supplemental material — Supplement methods. [file spectrum.01554-24-s0001.docx]

**Supplementary Methods**

1. **Nucleic Acids Extraction**

All blood samples were aseptically collected in EDTA-anticoagulated tubes and sent to the clinical laboratory for DNA extraction. We used commercial nucleic acid extraction reagents (S1006, Sansure, Changsha, China) and followed the supplier's protocol and previous study report (1, 2). Blood samples were processed as follows: 1 ml of blood was transferred to a sterile EP tube and centrifuged at 50×g for 10 minutes. After centrifugation, the upper plasma and white blood cell layer were transferred to a grinding tube and centrifuged at 12,000 rpm for 10 minutes. After discarding the supernatant, leaving 300 μl of the precipitate, 150 μl of lysis buffer and 30 μl of nucleic acid protectant were added to the tube, followed by vortexing. The sample was then homogenized using a tissue grinding homogenizer at 3,800 rpm for 30 seconds, with a 30-second interval, for a total of 2 cycles. After homogenization, the tube was centrifuged at 7,000 rpm for 3 minutes, and 20 μl of proteinase K was added. The tube was vortexed again and incubated at 65°C in a metal bath for 10 minutes. After incubation, 550 μl of liquid was carefully collected for the next step of automated magnetic bead nucleic acid extraction. Nucleic acid concentration and quality were quantified using a micro-volume spectrophotometer (Nanodrop 2000, Thermo, USA).

**2. NTS methodology and Bioinformatics methodology**

The standardized procedures for primer design, NTS amplification, sequence alignment, and bioinformatics analysis were provided by DGENSEE (WuHan, China), which have been detailed reported in previous studies (1-3).

**2.1 NTS Primer Design**

Complete gene sequences of the 16S rRNA and ITS1/2 from GenBank (accessed January 2019) were retrieved, with synthetic, lab-derived, and duplicate sequences manually removed. A maximum of 10 reference sequences per species were randomly selected, creating two distinct databases for 16S rRNA and ITS1/2. Multiple sequence alignment was performed using ClustalW (version 1.83) to assess base variation and identify conserved regions. Primer degeneracy was calculated using an in-house pipeline. The 27F/1492R primers for 16S rRNA and ITS1/ITS4 primers for ITS1/2 were chosen as the initial primers. Additional primers within 50 base pairs of these start primers were designed based on the following parameters: (1) primer length of 18–30 bp, (2) melting temperature (Tm) between 58–65°C with less than a 3°C difference between primers, (3) GC content of 40–60%, and (4) ΔG (Gibbs free energy) of the last five nucleotides at the 3' end being ≥-9 kcal/mol. Final primer pairs were formed by mixing the start and additional primers at a 3:1 molar ratio. The primers utilized in this study are provided in Supplementary Table 1, and all primer oligos were synthesized by Genscript.

**2.2 Amplification and Nanopore Targeted Sequencing**

To sequence multiple samples on a single chip, universal and specific primers were barcoded by attaching 96 unique barcode sequences to the 5' end. The barcode sequences were sourced from the Nanopore PCR barcode kit EXP-PBC096 (Oxford Nanopore Technologies). Amplification of the 16S rRNA gene and ITS1/2 was performed in a 20 µL reaction containing 8 µL of extracted DNA, 2 µL of primer (10 µM), and 10 µL of 2× Phusion U Multiplex PCR Master Mix (Thermo Fisher, USA). The thermocycling conditions were: 1 cycle at 98°C for 3 minutes, followed by 35 cycles at 98°C for 10 seconds, 55°C for 5 seconds, and 68°C for 10 seconds, with a final elongation at 68°C for 5 minutes. PCR products were purified using 0.8× AMpure beads (Beckman Coulter) and eluted in 10 µL of Tris-EDTA buffer. For the barcoded PCR, 5 µL of the eluate was combined with 5 µL of barcoded primers (10 µM) and 10 µL of 2× Phusion U Multiplex PCR Master Mix. The thermocycling conditions for barcoding were: 1 cycle at 98°C for 3 minutes, followed by 10 cycles at 98°C for 10 seconds, 55°C for 5 seconds, and 68°C for 5 seconds, with a final elongation at 68°C for 5 minutes. Barcoded products from 16S rRNA and ITS1/2 amplification were pooled at a 10:3:1 mass ratio. The pooled products from different samples were then combined to construct sequencing libraries using the 1D Ligation Kit (SQK-LSK109; Oxford Nanopore). Clinical samples, along with two extraction controls and two no-template controls, were included in a single sequencing library and sequenced using the Oxford Nanopore GridION X5 with real-time basecalling enabled.

- 1. **Bioinformatic methodology**

Basecalling and quality assessment of sequencing data were performed using the Oxford Nanopore GridION X5 and Guppy software in high-accuracy mode (ont-guppy-for-gridion v.1.4.3-1 and v.3.0.3-1). Sequencing reads shorter than 200 nt or longer than 2000 nt were excluded. An in-house script was utilized to analyze basecalling output, generating a real-time taxonomy list for each sample. The bioinformatics pipeline was initiated when every set of 4,000 reads passed the basecalling process. Adaptor trimming and barcode demultiplexing were conducted using Porechop (v.0.2.4) for reads that passed the basecalling step. The processed reads for each sample were then mapped to the 16S rRNA or ITS reference database (ftp://ftp.ncbi.nlm.nih.gov/refseq/TargetedLoci) using BLASTn (v.2.9.0+). Reads with more than 90% coverage were retained. Taxonomy assignments were made based on the taxonomic information of the mapped sequence. For reads assigned to the same species, a consensus sequence was generated using Medaka (v.0.10.1), which was then remapped to the 16S rRNA or ITS reference database. The highest scoring taxon was used as the final detection result for reads assigned to the same species. The taxonomy list, updated after each batch of 4,000 reads, was integrated into the existing taxonomy, providing an up-to-date list throughout the process.

**2.4 Standard strains and mock communities**

The ZymoBIOMICS Microbial Community DNA Standard (D6306, Cat No. ZRC190811) was sourced from Orange, California, USA. This mock community DNA contains genomic material from ten species, including eight bacterial and two fungal species: *Bacillus subtilis*, *Enterococcus faecalis*, *Escherichia coli*, *Lactobacillus fermentum*, *Listeria monocytogenes*, *Pseudomonas aeruginosa*, *Salmonella enterica*, *Staphylococcus aureus*, *Saccharomyces cerevisiae*, and *Cryptococcus neoformans*.

**2.5 Pathogen determination**

The NTS sequencing results were interpreted using algorithms designed to filter out taxonomically related microorganisms and apply specific criteria for pathogen detection. The detailed methodology for this process is outlined below: (1). Excluding closely related microorganisms. To reduce misalignments among closely related species, we applied penalties to the RPM (Reads Per Million) values for microorganisms within the same genus or family. Specifically, penalties of 10% for genus and 5% for family were implemented. For instance, if *Escherichia coli* registered an RPM of 100 and *Shigella sonnei* (belonging to the same Enterobacteriaceae family) had an RPM of 5, the RPM for *S. sonnei* would be adjusted to zero. (2). Criteria for pathogen detection involved using three types of negative controls to filter out potential contaminants from laboratory processes and human normal flora: "Long-termNC" (which includes a DNA extraction control and PCR Negative control monitored for 10 months, alongside 112 samples from healthy individuals), "DynamicNC" (negative controls collected within 30 days relative to each experimental sample's sequencing date, plus 112 samples from healthy individuals), and "BatchNC" (negative controls processed in the same sequencing batch). Each sample's sequences were normalized to the same sequencing depth of 10,000 reads. To identify potential laboratory contaminants, the abundance ratio of reads in a sample versus reads in the BatchNC, DynamicNC, or Long-termNC was calculated. These ratios were termed "Long-termNC-FoldChange" (LNC-FC), "DynamicNC-FoldChange" (DNC-FC), and "BatchNC-FoldChange" (BNC-FC), respectively. Furthermore, any strain detected in more than 50% of the NTC (No Template Control) samples was considered PCR contaminated. A list of clinically relevant pathogens was established based on a review of literature and clinical guidelines, alongside references to organisms in a pathogen database from published case reports(4). A read was counted if it matched within 50 bp upstream or downstream of the designated fragment. Mapping scores were assigned as follows: 1 for a sample-to-control ratio >10, 0.4 for a ratio between 3 and 10, and 0 for a ratio <3. A total mapping score between 1.2 and 2.4 indicated an inconclusive result, while a score below 1.2 was considered negative for infection.

**References**

1. Hong M, Peng D, Fu A, Wang X, Zheng Y, Xia L, Shi W, Qian C, Li Z, Liu F, Wu Q. 2023. The application of nanopore targeted sequencing in the diagnosis and antimicrobial treatment guidance of bloodstream infection of febrile neutropenia patients with hematologic disease. Journal of Cellular and Molecular Medicine 27:506-514.

2. Fu Y, Chen Q, Xiong M, Zhao J, Shen S, Chen L, Pan Y, Li Z, Li Y. 2022. Clinical Performance of Nanopore Targeted Sequencing for Diagnosing Infectious Diseases. Microbiology Spectrum 10:e0027022.

3. Zhang Y, Lu X, Tang LV, Xia L, Hu Y. 2023. Nanopore-Targeted Sequencing Improves the Diagnosis and Treatment of Patients with Serious Infections. mBio 14.

4. Blauwkamp TA, Thair S, Rosen MJ, Blair L, Lindner MS, Vilfan ID, Kawli T, Christians FC, Venkatasubrahmanyam S, Wall GD, Cheung A, Rogers ZN, Meshulam-Simon G, Huijse L, Balakrishnan S, Quinn JV, Hollemon D, Hong DK, Vaughn ML, Kertesz M, Bercovici S, Wilber JC, Yang S. 2019. Analytical and clinical validation of a microbial cell-free DNA sequencing test for infectious disease. Nature Microbiology 4:663-674.
