# Supplementary material for: Enhancing pathogens detection in suspected geriatric bloodstream infections using Nanopore-targeted sequencing
Source: Microbiol Spectr. 2024 Nov 22;13(1):e01554-24. doi: 10.1128/spectrum.01554-24 (PMC11705817; doi:10.1128/spectrum.01554-24)
Supplement: Table S1 — Primers for NTS used in this study. [file spectrum.01554-24-s0002.docx]

**Supplementary Table 1. Primers for NTS used in this study**

| Marker gene | Primer name | Primer sequence (Forward and Reverse) | Reference |
| --- | --- | --- | --- |
| 16s rRNA | 8F | Barcode sequence-GGATCCAGACTTTGATYMTGG | [1] |
|  | 27F | Barcode sequence-AGRGTTYGATYMTGGCTCAG | [1] |
|  | 38F | Barcode sequence-GGCTCAGRWYGAACGCTRG | [1] |
|  | 1492R | Barcode sequence-RGYTACCTTGTTACGACTT | [1] |
|  | 1495R | Barcode sequence-TASRGYTACCTTGTTACGA | [1] |
| ITS1/2 | ITS1 | Barcode sequence-TCCGTAGGTGAACCTGCGG | [1] |
|  | ITS1-2 | Barcode sequence-GTGAACCTGCGGAAGGATCAT | [1] |
|  | ITS4 | Barcode sequence-TCCTCCGCTTATTGATATGC | [1] |
|  | ITS4-2 | Barcode sequence-TATGCTTAAGTTCAGCGGGT | [1] |
|  | ITS1-barcode | Barcode sequence-TCCGTAGGTGAACCTGCGG | [2] |
|  | ITS1-2-barcode | Barcode sequence-GTGAACCTGCGGAAGGATCAT | [2] |
|  | ITS4-barcode | Barcode sequence-TCCTCCGCTTATTGATATGC | [2] |
|  | ITS4-2-barcode | Barcode sequence-TATGCTTAAGTTCAGCGGGT | [2] |
| rpoB | MF | Barcode sequence-CGACCACTTCGGCAACCG | [2] |
|  | MR | Barcode sequence-TCGATCGGGCACATCCGG | [2] |
|  | MF-2 | Barcode sequence-GACGACATCGACCACTTCGG | [2] |
|  | MR-2 | Barcode sequence-GGGTCTCGATCGGGCACAT | [2] |
